# Treatment of Diphtheria

**Published:** 1866-07

**Authors:** 


Treatment of Diphtheria.—Dr. Greenhow, well known as an authority on this subject, makes known his most recent experience in regard to the treatment of diphtheria, in a clinical lecture reported in the London *Lancet.* In his estimation, the most valuable of all remedies in the asthenic form of the disease, is the tincture of iron (tinc. ferri chloridi, U.S.P.) in full doses, proportioned to the age of the patient. In milder cases he uses the chlorate of potassa both as a gargle and internally. As far as we can judge, these medicines are in very general use by American practitioners. We are in the practice of directing the patient to retain the medicine, whether the iron or the chlorate, for some moments in the fauces before swallowing, so as to get the benefit of their local application. An excellent method of using the chlorate for children is to powder the salt finely with an equal quantity, or twice the quantity, of sugar, and put it in the mouth frequently, a pinch at a time. This mode of administering it is very beneficial in many throat affections of adults.—*Pacific Med. & Surg. Jour.*

